# Meta-analytic methods for neuroimaging data explained

**DOI:** 10.1186/2045-5380-2-6

**Published:** 2012-03-08

**Authors:** Joaquim Radua, David Mataix-Cols

**Affiliations:** 1 Institute of Psychiatry, King's College London, De Crespigny Park, London, UK; 2 Research Unit, FIDMAG-CIBERSAM, Sant Boi de Llobregat, Barcelona, Spain

**Keywords:** activation likelihood estimation, effect-size signed differential mapping, functional magnetic resonance imaging, kernel density analysis, meta-analysis, magnetic resonance imaging, multilevel kernel density analysis, parametric voxel-based meta-analysis, region of interest, signed differential mapping, voxel-based morphometry

## Abstract

The number of neuroimaging studies has grown exponentially in recent years and their results are not always consistent. Meta-analyses are helpful to summarize this vast literature and also offer insights that are not apparent from the individual studies. In this review, we describe the main methods used for meta-analyzing neuroimaging data, with special emphasis on their relative advantages and disadvantages. We describe and discuss meta-analytical methods for global brain volumes, methods based on regions of interest, label-based reviews, voxel-based meta-analytic methods and online databases. Regions of interest-based methods allow for optimal statistical analyses but are affected by a limited and potentially biased inclusion of brain regions, whilst voxel-based methods benefit from a more exhaustive and unbiased inclusion of studies but are statistically more limited. There are also relevant differences between the different available voxel-based meta-analytic methods, and the field is rapidly evolving to develop more accurate and robust methods. We suggest that in any meta-analysis of neuroimaging data, authors should aim to: only include studies exploring the whole brain; ensure that the same threshold throughout the whole brain is used within each included study; and explore the robustness of the findings via complementary analyses to minimize the risk of false positives.

## Introduction

The number of neuroimaging studies has grown exponentially in recent years. However, findings from different studies may sometimes be difficult to integrate into a coherent picture. Inconsistent results are not uncommon. Furthermore, a few influential studies might often eclipse robust findings from other studies. In other words, we may at times not see the forest for the trees. In this context, meta-analyses are helpful to combine and summarize the data of interest and potentially offer insights that are not immediately apparent from the individual studies.

The present paper aims to describe the main methods which have been used for the meta-analysis of neuroimaging data, as well as their advantages and drawbacks, with some examples of application to mood and anxiety disorders. The first section of the paper introduces how a standard meta-analysis is conducted, that is, when there is only one variable of interest, with an example from a meta-analysis of global brain volumes. This is important for a better appreciation of the pros and cons of the meta-analytic methods that we review later. The second section of the paper describes the meta-analyses of neuroimaging studies based on regions of interest (ROI) and their particular issues. The third section introduces the various available voxel-based meta-analytic methods, which aim to overcome some of the limitations of the ROI-based methods but have, in turn, their own limitations. The similarities and differences between the various voxel-based methods are also discussed in depth. Finally, we describe the available online databases of neuroimaging studies. This paper is meant to be accessible for the applied researcher in the fields of psychiatry, neurology and allied disciplines. Other excellent, more technical, reviews of meta-analytical methods can be found elsewhere [1,2].

### Standard meta-analyses

Prior to any meta-analytic calculation, researchers conduct an exhaustive and critical literature search, often including contact with the authors of the original studies in order to retrieve important pieces of missing information. Then, researchers conduct a mathematical summary of the findings of the included studies (that is, the meta-analysis proper). Finally, researchers apply a series of tests, plots and subgroup analyses to assess the heterogeneity and robustness of the results. The latter step, along with the exhaustive and critical inclusion of studies, is of utmost importance in order to obtain unbiased meta-analytic conclusions.

With the aim of introducing the reader to the logics of a standard meta-analysis, in this section we will use as an example a meta-analysis of global gray matter volumes in patients with obsessive-compulsive disorder (OCD) (see Table 1). The included studies correspond to seven publications reporting global gray matter volume, which were included in a published meta-analysis of voxel-based morphometry studies in OCD [3].

**Table 1:** Global gray matter volumes reported in seven studies on obsessive-compulsive disorder

	Reference	Patients		Controls		Patients + controls		Difference		Effect size	
		
		Number	Volume ± SD	Number	Volume ± SD	N	Variance	Estimate	Variance	Estimate	Variance
Study 1	[46]	18	773 ± 56	18	822 ± 56	36	3,114	-49	346	-0.854	0.122
Study 2	[47]	55	685 ± 74	50	708 ± 72	105	5,323	-23	203	-0.313	0.039
Study 3	[48]	25	850 ± 83	25	834 ± 71	50	5,997	+16	480	0.196	0.080
Study 4	[49]	72	739 ± 82	72	763 ± 78	144	6,404	-24	178	-0.298	0.028
Study 5	[50]	37	776 ± 69	26	747 ± 68	63	4,680	+29	307	0.418	0.067
Study 6	[51]	19	827 ± 44	15	836 ± 63	34	3,041	-9	363	-0.179	0.120
Study 7	[52]	71	740 ± 66	71	738 ± 63	142	4,119	+2	116	0.035	0.028

All volumes are in milliliters. SD: standard deviation.

#### Weighting of the studies

In order to summarize these seven studies, a simple meta-analysis could consist of calculating the mean difference in global gray matter volume between patients and controls as reported in the original studies [4]. Thus, we could summarize Table 1 by saying that the mean global gray matter volume is 8.4 mL inferior in patients than in healthy controls-this number is just the arithmetic mean of the differences shown in the table.

The use of the arithmetic mean, however, may be too simplistic, because the different studies should have different weights. For example, the number of patients in study 4 is four times larger than the number of patients in study 1. Clearly, we cannot give the same weight to both studies and should give more weight to study 4. Probably, we should give it about four times more weight, as it includes four times as many patients.

Including all the studies in Table 1 and weighting the mean difference by the sample sizes of the studies, we would conclude now that the mean global gray matter volume is 8.8 mL inferior in patients than in controls. Note that when we previously calculated the mean difference as the simple arithmetic mean, we were indeed assuming that all the studies had the same sample size. This erroneous assumption had only a minor effect here (we thought that the difference was about 5% smaller than what we think now), but it could have important effects in other meta-analyses, especially if studies with smaller sample sizes inexplicably find more differences than studies with larger sample sizes-we will introduce how to detect this kind of bias later.

Unfortunately, weighting the calculations only by sample size would still be too simplistic, because the weight of a study should also include its precision. For example, study 1 included fewer patients than study 4, but its volume estimates seem much more precise, as its sample variance is approximately the half than that in study 4 (see Patients + controls column in Table 1). We do not know the reason for this higher precision (maybe the sample was more homogenous; maybe the technical procedures were cleaner; maybe it was just chance); however, we must take this precision into account by weighting by the inverse of the variance of the difference-which also includes the sample size.

Including all the studies in Table 1 and weighting by the inverse of the variance of the difference, we would conclude that the mean global gray matter volume is 8.9 mL inferior in patients than in controls (z-value = -1.55, *P *= 0.121). When previously we did not weight by sample variance we were assuming that all the studies had the same variance, though in this case this assumption was acceptable because the variance of the studies is rather homogeneous.

However, as explained in the next section, weighting the calculations only by the inverse of the variance of the difference may still be too simplistic.

#### Heterogeneity in the studies

Healthy individuals have different global gray matter volumes, that is, some have larger brains, some have thicker cortices, and so on. When conducting an analysis with original data, we are usually able to explain or model a part of this variability, but there is also a part of this variability that remains unexplained. This residual error may be due to unobserved variables, etiological heterogeneity within particular diagnoses, poor model fitting, or maybe just pure chance. This individual-based within-study variability cause the sample means to be variable, so that different studies obtain different results.

However, within-study variability is not the only source of the between-study variability or heterogeneity. Given the relatively small amount of robust findings in neuroimaging, it would be highly desirable that all researchers conducted their studies using the exact same inclusion criteria and methods so that all between-study variability was only related to the within-study variability. However, the fact is that clinical and methodological differences between studies are often substantial.

On the one hand, patients included in the individual studies may have been sampled from clinically different populations; for example, one study of major depressive disorder may include outpatients with mild reactive depressive episodes while another study may be focused on inpatients suffering from severe endogenous depressions with melancholic symptoms. Similarly, patients in different studies may be receiving different treatments, or be in different phases of the disorder (for example, having a first episode or having a history of multiple episodes).

On the other hand, researchers may have been investigated similar but still different aspects of a disorder; for example, one study may have described the blood oxygen level-dependent (BOLD) brain response to a task involving a high memory load, while another study may be interest in the BOLD response to a task related to decision-making. Or even if studying the same particular cognitive function, each study may employ a particular statistical package, and its large set of associated assumptions.

Finally, there may be a relevant part of the between-study heterogeneity which can be neither related to the within-study variability, nor explained by clinical or methodological differences between studies. This is called residual heterogeneity.

It is highly recommended to study the between-study variability or heterogeneity in any meta-analysis. For example, if the main analysis detects differences between patients and controls, it may be of interest to explore whether these differences depend on the severity of the disorder, or if they are related to special subtypes of the disorder. These questions may be assessed with meta-regressions. But even if the meta-analysis does not aim to explore the modulating effects of clinical variables on the main outcomes, heterogeneity should still be taken into account.

**Figure 1:**
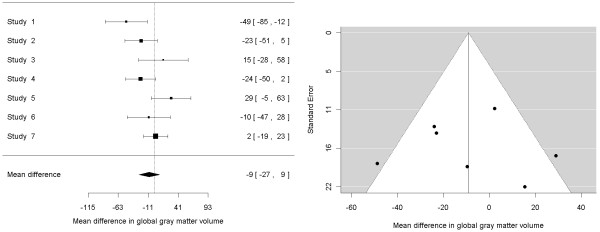
**Forest (left) and funnel (right) plots of the mean differences in global gray matter volume between patients with obsessive-compulsive disorder and healthy controls (using a random-effects model)**. On the funnel plot, the included studies appear to be symmetrically distributed on either side of the mean difference, suggesting no publication bias towards positive or negative studies.

Indeed, there is agreement on always including the residual heterogeneity in the weighting of the calculations [5-8]. Meta-analyses conducted this way are said to follow random-effects models, in opposition to the fixed-effects models that we saw in the previous point, which did not include heterogeneity. In the example of Table 1 the use of a random-effects model would lead us to conclude that the mean global gray matter volume is 9.0 mL inferior in patients than in controls (z-value = -0.98, *P *= 0.328; see Figure 1, left). Note the increase of *P*-value in the random-effects model (from 0.121 to 0.328), thus better controlling the false positive rate.

#### Other complementary analyses

The above meta-analysis of global gray matter volumes in OCD was only aimed to help interpret the findings of a regional voxel-based meta-analysis, for which no other tests beyond a correctly weighted random-effects model would probably be required. However, if global gray matter volumes were the main outcome of a meta-analysis, some complementary plots and tests would be recommended to help the reader assess the reliability and robustness of the findings [9].

On the one hand, the meta-regressions described above may be useful for assessing if the findings are predominantly (or only) present in one group of patients, for example, in those with more severe forms of OCD. In this regard, specific meta-analyses of subgroups of patients may further confirm these hypotheses or, also important, may state that the abnormalities are present in all subgroups of patients, increasing the robustness of the findings. Similarly, sensitivity analyses consisting of repeating the meta-analysis many times, each time with a different combination of studies, may be useful to assess whether the findings are driven by one or few studies. Finally, funnel plots (see Figure 1, right) may be useful for appraising whether studies with small samples report more statistically significant findings than studies with larger samples. This is typical of subjects with publication bias, where studies with small samples are only published if their results match *a priori *hypotheses.

It is important to note that these kinds of tests and graphical aids are necessary but do not provide conclusive information, and should only be interpreted in the context of the field under investigation. A symmetrical funnel plot, for example, is not an amulet against publication bias, especially in some types of meta-analysis. ROI-based studies, for instance, may be more prone to be affected by publication biases, as the authors may decide which brain regions are reported and which are not. Conversely, an asymmetrical funnel plot would not necessarily invalidate a meta-analysis if publication bias appears unlikely. This may be the case of voxel-based studies, where the whole brain is included in the analysis.

#### Use of effect sizes

Most meta-analyses do not use the raw volume differences as we exemplified in the previous points, but rather, they use standardized volume differences, that is, effect sizes [10]. Briefly, a raw difference is the difference in milliliters between patients' and controls' global gray matter volume, while a standardized difference is the difference is standard deviations-usually corrected for small sample size bias.

**Figure 2:**
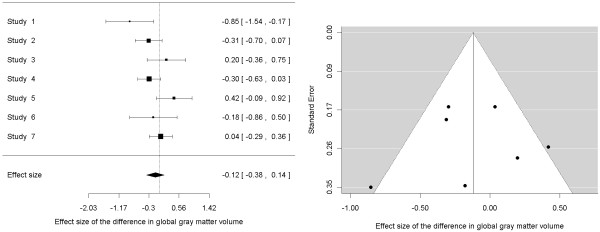
**Forest (left) and funnel (right) plots of the effect size of the differences in global gray matter volume between patients with obsessive-compulsive disorder and healthy controls (using a random-effects model)**. On the funnel plot, the included studies appear to be symmetrically distributed on either side of the mean effect size, suggesting no publication bias towards positive or negative studies.

This subtle difference has a series of consequences. First, the unit of measure (milliliters, in this case) is lost, which makes the interpretation of the findings less straightforward but indeed more comparable with other measures, for example, an effect size of d = 0.5 is considered 'medium' independently of whether it refers to a difference in gray matter volume, in BOLD response or in a questionnaire score. Using the data from Table 1, the effect size of the difference in global gray matter volume between patients and controls is d = -0.122 (z-value = -0.93, *P *= 0.354; Figure 2), which is below the conventional range of 'small' effect (0.2 to 0.3) [11]. Second, a study reporting a larger difference may be found to have a smaller effect size, or vice versa, depending on the sample variance. For instance in Table 1, the raw difference is slightly larger in study 4 than in study 3, whilst the effect size is slightly larger in study 3 than in study 4. Third, and very important, effect size can be directly derived from many statistics like a *t*-value or a *P*-value, which are much more often reported than sample statistics; that is, we can often know the effect size but not the raw difference. This advantage usually allows a much more exhaustive inclusion of studies, thus clearly justifying the use of effect sizes in many meta-analyses.

### Meta-analyses based on regions of interest

A ROI is a part of the brain that the authors of the study wish to investigate, usually based on *a priori *hypotheses. ROI-based studies usually select a set of few ROIs and manually delimitate them on the raw images. Researchers then analyze the volume of these ROIs, their mean BOLD response to a stimulus, their positron emission tomography ligand volume of distribution, or any other measure of interest.

#### Region of interest-based meta-analyses

A typical ROI-based meta-analysis can be viewed as a set of different meta-analyses, each of them applied to a different ROI. These meta-analyses can usually be optimally conducted with all appropriate weightings and complementary analyses, as seen for example in the meta-analysis of regional brain volumes in OCD conducted by Rotge *et al. *[12], in which the analyses are based on effect sizes and random-effects models and complemented with explicit assessments of the heterogeneity, several sensitivity analyses, funnel plots and meta-regressions. Unfortunately, each original study included in this meta-analysis only investigated a small set of brain regions, causing the meta-analyses to include only a very small number of studies for each brain region. Indeed, only three or four studies could be included for highly relevant regions such as the putamen or the anterior cingulate cortex, both of which were found as abnormal in subsequent voxel-based meta-analyses [3,13]. Other brain regions could not be meta-analyzed because they had been investigated by too few or no studies. Needless to say, this would not be the case for those ROI studies reporting whole brain results in online supplements or similar, but this is seldom the case.

Moreover, it must be noted that some brain regions are more frequently studied than others, which causes the statistical power to differ depending on the brain region under study. In the example, while data from five studies or more were available for the orbitofrontal cortex, the thalamus and the caudate nuclei, some brain regions could not be meta-analyzed at all.

Ultimately, the authors of the original studies have a set of *a priori *hypotheses which influence their decision to investigate differences in a given brain region at the expense of other regions. These decisions determine the number of studies investigating that brain region, and thus the statistical power to detect that brain region as significantly different between patients and controls in a ROI-based meta-analysis. Publication bias is also a problem as studies failing to report statistically significant differences on hypothesized ROIs may be less likely to ever be become publicly available. A recent analysis of more than 450 ROI-based neuroimaging studies in psychiatry illustrates this point well [14]. The author demonstrated that the number of studies reporting significant results was nearly the double than expected, suggesting strong publication biases in the ROI literature.

Another consideration is the heterogeneous definition or the boundaries of the ROIs, which may differ from one study to the other [12]. However, this variability might have a relatively small impact on effect sizes, as boundary definitions are the same for the patients and controls included in a study. Furthermore, the spatial error may probably be counteracted by the higher anatomical accuracy achieved by the manual delimitation of the ROIs in the original studies [15,16].

#### Label-based reviews

Some authors have used a simplified type of ROI-based meta-analysis, consisting of just counting how many times a particular ROI is detected as significantly abnormal in patients versus healthy controls. This procedure has been called label-based review [17]. For example, in their functional neuroimaging meta-analysis of the brain's response to emotional tasks, Phan *et al. *[18] represented each activation peak as a dot in an atlas of the brain. They then divided the brain into 20 ROIs and counted how many studies had one or more activation peak in each ROI.

**Figure 3:**
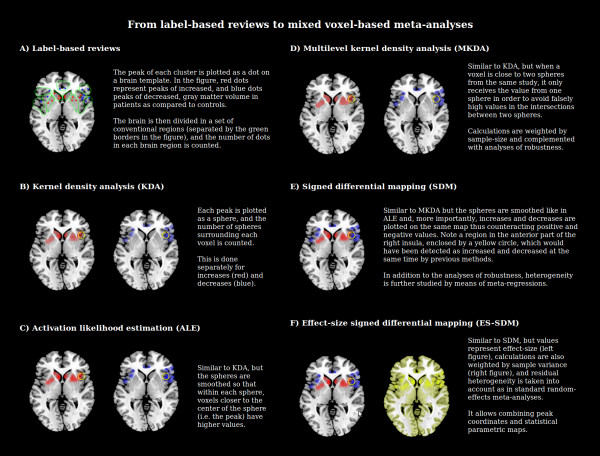
**Summary of the main available voxel-based meta-analytic methods**. Increases and decreases of gray matter volume are fictitious and have been manually plotted over a MRICroN template to illustrate the main features of the different methods.

A fictitious example of such approach is shown in Figure 3A. Here, the studies would have reported that the patients with mood or anxiety disorders have increases of gray matter volume in the basal ganglia, extending to the anterior part of the right insula, as well as decreases of gray matter volume in the anterior cingulate and insular cortices. The authors of a label-based review would have first plotted the peaks of the clusters of significant increase (red) or decrease (blue) of gray matter volume in a brain template. Then, they would divide the brain into several regions, for example, anterior cingulate gyrus, left and right inferior frontal gyri, insulas, superior temporal gyri, caudate nuclei, putamen nuclei, and so on. Finally, they would have counted how many peaks lay within each of these regions.

This method may be useful when other approaches are not feasible, for example when not enough information is available for conducting a ROI-based or a voxel-based meta-analysis. Its simplicity, however, may conceal a series of important drawbacks which must be taken into account. First, no weighting of the studies is performed, which means that all studies are assumed to have the same sample size and precision. This is a strong and unrealistic assumption which may be violated in most meta-analysis. Fortunately, sample size information is always available, and so label-based meta-analyses should at least be weighted by sample size. Second, the findings of the studies are binarized (significant versus not-significant), leading to a loss of information on the magnitude of the raw differences or on the effect sizes. Third, it is not clear whether studies reporting opposite findings in a particular ROI (for example, volume decrease in some studies and volume increase in others) are adequately dealt with. Finally, they may be also affected by the particular issues of ROI-based meta-analyses described above.

### Voxel-based meta-analyses

Scanner three-dimensional images are composed of thousands of tiny cubes (or rectangular cuboids) called voxels, in the same way that digital photographs are composed of thousands of tiny squares called pixels. Voxel-based methods consist of conducting the meta-analytic calculations separately in each voxel of the brain, thus freeing the meta-analysis from aprioristic anatomical definitions. There are different types of voxel-based meta-analyses, including image-based, coordinate-based and mixed image- and coordinate-based meta-analyses.

#### Image-based meta-analyses

An image-based meta-analysis should be understood as a voxel-based version of the standard meta-analysis, that is, it consists of thousands of standard meta-analyses, each of them applied to a different voxel [2,19]. The data of each study is retrieved from its statistical parametric maps (the three-dimensional images resulting from the comparison between patients and controls), and thus include the whole brain. This technique shares some limitations with any voxel-based analysis, mainly relating to the massive number of statistical tests (that is, one test for each voxel). The correction of multiple comparisons is an unsolved issue, with current methods being either too liberal or too conservative [19]. For this reason, thresholds based on uncorrected *P*-values and cluster-size are usually preferred [1,19,20]. Also, such massive-scale testing prevents a careful visual inspection of the analyses (for example, to describe relevant non-significant trends).

However, the biggest drawback of image-based meta-analyses is that the statistical parametric maps of the original studies are seldom available, therefore seriously limiting the inclusion of studies.

#### Coordinate-based meta-analyses

Given the poor availability of statistical parametric maps, early meta-analyses of voxel-based studies consisted of label-based (rather than image-based) reviews, as discussed earlier. These methods quickly evolved to coordinate-based meta-analyses, which in their simplest form consisted of counting, for each voxel, how many activation peaks had been reported within its surroundings [21]. In the fictitious example, the dots of the label-based review (Figure 3A) would be replaced with spheres (Figure 3B, C), and the brain would not be divided into conventional regions but rather the number of spheres surrounding each voxel would be counted, thus obtaining a count for each voxel. It must be noted that calculations in activation likelihood estimation (ALE) [22] are not exactly based on counting the number of spheres but on computing the probability of a union, though in practice, the latter behaves like the former.

The use of voxels rather than conventional divisions of the brain improved the anatomical localization of the findings. However, the first available methods, namely ALE and kernel density analysis (KDA) [21], had some additional issues which enlarged the list of drawbacks of label-based reviews. Specifically, they only counted the total number of peaks, independently of whether they came from the same or different studies, and thus the analysis could not be weighted by sample size and a single study reporting many peaks in close proximity could drive the findings of the whole analysis.

These drawbacks led to the creation of a second generation of coordinate-based meta-analytic methods, mainly evolved versions of KDA, such as multilevel KDA [23] and parametric voxel-based meta-analysis [24]; evolved versions of ALE [25,26]; and signed differential mapping (SDM) [3,27-30] (Figure 3D, E), which overcame these limitations by separating the peaks of each study. Moreover, some of these new methods weighted the studies by their sample size and included a series of complementary analyses to assess the reliability and robustness of the findings. One of the methods, SDM, also addressed between-study heterogeneity by reconstructing positive and negative maps in the same image, thus counteracting the effects of studies reporting findings in opposite directions [31] and incorporating meta-regression methods. Finally, SDM included templates for white matter [32], allowing multimodal meta-analyses which were not possible with previous methods [33]. However, these methods still did not employ the standard statistical methods of the ROI-based meta-analyses, for example, they did not weight by the precision of each study.

Several of these methods have recently been applied to mood and anxiety disorders. One such was a meta-analysis of studies investigating bipolar disorder [34] using SDM, which found patients to have gray matter reductions in the left medial frontal and/or anterior cingulate cortex (MF/ACC) and bilateral anterior insula, with complementary analyses showing findings in the left ACC and right insula to be robust, and left MF/ACC volume to be higher in samples where patients were being treated with lithium. An SDM meta-analysis of studies investigating major depressive disorder also found patients to have gray matter reductions in the MF/ACC [35], and complementary analyses showed this finding to be robust and more severe in multiple-episode samples. Recent SDM meta-analyses of studies investigating anxiety disorders have also found patients to have gray matter reduction in the MF/ACC [13], along with abnormalities in the basal ganglia. Specifically, patients with OCD were found to have increased gray matter volume in the bilateral putamen and caudate nuclei [3], with complementary analyses showing findings in the MF/ACC and left basal ganglia to be robust, and bilateral increases of basal ganglia volume to be higher in samples including patients with higher symptom severity. Conversely, patients with anxiety disorders other than OCD were found to have decreased gray matter volume in the left putamen nucleus [13].

An example of the use of these methods in functional neuroimaging is the recent study by Delvecchio *et al. *[36], which meta-analyzed the functional brain response to emotional faces using ALE. They found that both patients with bipolar disorder and patients with unipolar depression displayed limbic hyperactivations. However, only patients with bipolar disorder showed a set of hypoactivations in the prefrontal cortex and hyperactivations in the thalamus and basal ganglia. Conversely, only patients with major depressive disorder showed hypoactivations in the sensorimotor cortices. Another example from the anxiety disorders literature is a multilevel KDA meta-analysis, which found that patients showed hyperactivations in the amygdala and insula [37]. Interestingly, these hyperactivations were more observed in phobias, whilst patients with post-traumatic stress disorder displayed a hypoactivation of the MF/ACC. Finally, an ALE meta-analysis of functional differences in patients with OCD detected that symptom provocation was possibly associated with activation of the orbitofrontal cortex, prefrontal cortex, anterior cingulate cortex, precuneus, premotor cortex, superior temporal gyrus, basal ganglia, hippocampus and uncus [38].

These methods may also be applied to functional connectivity studies [39,40]. Laird *et al*., for instance, studied the default mode network by first creating ALE maps, and then deriving meta-analytic co-activation maps [40]. With this approach, they could identify an affective sub-network component. This approach is promising, given the increasing interest in functional connectivity studies in various mood and anxiety disorders.

#### Mixed image- and coordinate-based meta-analyses

Recently, effect size SDM (ES-SDM) was designed to allow the combination of studies from which images (statistical parametric maps) are available with studies from which only peak coordinates are reported, thus allowing a more exhaustive inclusion of studies, as well as more accurate estimations [19].

This is achieved by first using peak coordinates and their statistical values to recreate the statistical parametric maps, and then conducting an image-based meta-analysis. Thus, this method has some statistical advantages as compared to previous coordinate-based methods, namely the use of standard statistical methods (for example, weighting the calculations by both sample size and study precision, use of effect sizes, inclusion and assessment of residual heterogeneity, and so on).

In a meta-analysis of the BOLD response to emotional facial stimuli [19], the sensitivity to detect real activations (that is, the number of actually activated voxels appearing as significant in the meta-analysis, divided by the total number of actually activated voxels) was similar between ES-SDM (55%) and SDM (51%) when only using peak coordinates. However, the inclusion of the statistical parametric maps led to a gradual and substantial increase of the sensitivity of ES-SDM (73% when the statistical parametric map of one study was included, 87% when the statistical parametric maps of two studies were included, 93% when the statistical parametric maps of three studies were included, and so on). Therefore, given the potential of this new method, we would encourage authors to make their statistical parametric maps widely available to the community on their laboratory websites or via other means.

### Online databases

In parallel with the development of new meta-analytical methods, several freely-available website-based databases of neuroimaging data have been made available. These online databases may be classified in three groups, namely: sets of original data (for example, the raw scanner images from several samples of individuals); summary statistics from the studies included in one meta-analysis (for example, the mean ± standard deviation ROI volumes); and sets of summary statistics of virtually all published studies.

The online sets of original data are composed of the raw and/or pre-processed brain images, along with the demographic and clinical characteristics of each of the many anonymous participants. These databases may be used by researchers to conduct their studies, thus being a useful resource for highly accurate data analyses. It must be noted, however, that analyses derived from these datasets should not be strictly considered meta-analyses, as they do not necessarily exhaustively include all available data. Examples of these datasets are BRAINNet (http://www.brainnet.net), the fMRI Data Center (http://www.fmridc.org) and OpenfMRI (http://www.openfmri.org).

Online databases containing the summary statistics from the studies included in particular meta-analyses represent a more interactive (and often complete) alternative to the traditional 'supplementary materials' that accompany published meta-analyses. Importantly, these online data may be used by other researchers to conduct updated or secondary analyses. Examples of this type of databases are the Bipolar Disorder Neuroimaging Database (http://www.bipolardatabase.org) and the Major Depressive Disorder Neuroimaging Database (http://www.depressiondatabase.org) by Kempton and colleagues [41,42], as well as the series of peak-coordinate databases from SDM meta-analyses (http://www.sdmproject.com/database).

Finally, many sets of summary statistics of virtually all published neuroimaging studies exist, allowing a rapid retrieval of specific data in order to facilitate the meta-analytic process. The developers of BrainMap (http://www.brainmap.org), for instance, have been building and updating an impressive database of neuroimaging findings since 1987 [43]. Other available databases are the AMAT toolbox (http://www.antoniahamilton.com/amat.html), the Brede Database (http://neuro.imm.dtu.dk/services/brededatabase) [44], the Internet Brain Volume Database (http://www.cma.mgh.harvard.edu/ibvd) and the Surface Management System Database (http://sumsdb.wustl.edu/sums/index.jsp).

Another recent and promising online development called NeuroSynth (http://www.neurosynth.org) [45] deserves mentioning. NeuroSynth contains a set of summary statistics together with online functions aimed to conduct real-time meta-analyses online. Unfortunately, extraction of coordinates from publications is not manually verified, which may bias the results towards those regions that the authors of the original articles wanted to emphasize in the tables of the manuscripts. However, when the researcher's goal is to obtain a very fast and preliminary meta-analysis of the literature, NeuroSynth may be one of the first options.

## Conclusions

In this paper we have reviewed the main types of meta-analytic methods available for neuroimaging studies, using examples from the mood and anxiety disorder literature to illustrate these methods.

ROI- and voxel-based methods each have advantages and disadvantages, which are summarized in Table 2. Specifically, ROI-based meta-analyses use optimal statistical methods, but they usually have a limited and likely biased inclusion of studies [14]. Conversely, voxel-based meta-analyses usually have a more exhaustive and unbiased inclusion of studies, but their statistical methods are less accurate. There are also relevant differences between the different available voxel-based meta-analytic methods. Fortunately, the field is rapidly evolving to develop more accurate and robust methods.

**Table 2:** Comparison of the main meta-analytic methods for neuroimaging studies comparing patients and controls

	**ROI-based meta-analyses**	**Voxel-based meta-analyses**			
		
		**KDA/old ALE**	**Multilevel KDA/new ALE**	**SDM**	**ES-SDM**
	
**Selection of studies**					
Exhaustive inclusion of studies	Limited, as information for a given brain region is present in few or no studies	Probable, as far as the included studies investigate the whole brain and not only some ROIs (in which case should be discarded)			More probable, because statistical parametric maps can also be included
Unbiased inclusion of studies	Limited, as information is only available for regions hypothesized *a priori*, ignoring the rest of the brain	Probable, as far as the included studies do not use different statistical thresholds for different parts of the brain (this is a strict inclusion criterion in SDM and ES-SDM)			
**Statistical analyses**					
Weighting of the studies	Complete (sample size and study precision)	None	Partial (only sample size)		Complete (sample size and study precision)
Control of the heterogeneity	Residual heterogeneity is correctly included in the analyses	Residual heterogeneity is not controlled, and increases and decreases are not counteracted, potentially leading to voxels being detected as increased and decreased at the same time		Residual heterogeneity is not included in the weightings, but increases and decreases are counteracted	Residual heterogeneity is correctly included in the weightings
Study of the heterogeneity	Possible, by means of meta-regressions and subgroup analyses	Limited to subgroup analyses		Possible, by means of meta-regressions and subgroup analyses	
Correction for multiple comparisons	Possible	Not possible, questionable or limited to conventional voxel-thresholded cluster-based methods			
Description of the effect sizes	Possible	Not possible	Possible though limited to pseudo-effect sizes based on the proportion of studies reporting significant findings		Possible
Description of relevant non-significant trends	Possible, as the number of ROIs is manageable	Not possible, or limited to the visual inspection of liberally thresholded maps, as the number of voxels is too massive for a more accurate individual inspection			

Please see text for further details. ALE: activation likelihood estimation; ES: effect size; KDA: kernel density analysis; ROI: region of interest; SDM: signed differential mapping.

Although voxel-based meta-analyses minimize the effects of selectively reporting certain ROI, they are not totally immune to publication biases, as negative results may still be less likely to be published (what is known as the file drawer problem). Authors of the original papers are strongly encouraged to publish their results even if they perceive them as being disappointing or they do not find differences between patients and controls.

Finally, we suggest that in any meta-analysis of neuroimaging data, independently of the chosen method, authors should aim to: only include studies which explored the whole brain; ensure that the same threshold throughout the whole brain was used within each included study; and explore the robustness of the findings with several complementary analyses, for example, sensitivity analyses, quantification of the ROI- or voxel-based between-study heterogeneity [19], funnel plots of the values extracted from the meta-analytic clusters or their peaks, and so on, just like in any standard meta-analysis.

## Competing interests

The authors declare that they have no competing interests.

## Authors' contributions

All authors drafted, read and approved the final manuscript.
